# The Pleiotropic Effects of the Canonical Wnt Pathway in Early Development and Pluripotency

**DOI:** 10.3390/genes9020093

**Published:** 2018-02-14

**Authors:** Anchel de Jaime-Soguero, Willy Antoni Abreu de Oliveira, Frederic Lluis

**Affiliations:** Stem Cell Signalling Laboratory, Department of Development and Regeneration, KU Leuven Stem Cell Institute, Herestraat 49, Onderwijs en Navorsing 4, Box 804, 3000 Leuven, Belgium; Anchel.deJaimeSoguero@kuleuven.be (A.d.J.-S.); willyantoni.abreudeoliveira@kuleuven.be (W.A.A.d.O.)

**Keywords:** Wnt/β-catenin pathway, embryonic stem cells, pre-implantation development, cell cycle, somatic cell reprogramming

## Abstract

The technology to derive embryonic and induced pluripotent stem cells from early embryonic stages and adult somatic cells, respectively, emerged as a powerful resource to enable the establishment of new in vitro models, which recapitulate early developmental processes and disease. Additionally, pluripotent stem cells (PSCs) represent an invaluable source of relevant differentiated cell types with immense potential for regenerative medicine and cell replacement therapies. Pluripotent stem cells support self-renewal, potency and proliferation for extensive periods of culture in vitro. However, the core pathways that rule each of these cellular features specific to PSCs only recently began to be clarified. The Wnt signaling pathway is pivotal during early embryogenesis and is central for the induction and maintenance of the pluripotency of PSCs. Signaling by the Wnt family of ligands is conveyed intracellularly by the stabilization of β-catenin in the cytoplasm and in the nucleus, where it elicits the transcriptional activity of T-cell factor (TCF)/lymphoid enhancer factor (LEF) family of transcription factors. Interestingly, in PSCs, the Wnt/β-catenin–TCF/LEF axis has several unrelated and sometimes opposite cellular functions such as self-renewal, stemness, lineage commitment and cell cycle regulation. In addition, tight control of the Wnt signaling pathway enhances reprogramming of somatic cells to induced pluripotency. Several recent research efforts emphasize the pleiotropic functions of the Wnt signaling pathway in the pluripotent state. Nonetheless, conflicting results and unanswered questions still linger. In this review, we will focus on the diverse functions of the canonical Wnt signaling pathway on the developmental processes preceding embryo implantation, as well as on its roles in pluripotent stem cell biology such as self-renewal and cell cycle regulation and somatic cell reprogramming.

## 1. Introduction

Stem cells have, by definition, the ability to self-renew, i.e., give rise to at least one identical daughter cell, as well as to differentiate into the myriad of specialized cell types from early embryonic stages of development until adult age. These hallmark characteristics of stem cells have sparked a widespread interest in their biology, which fueled formidable research efforts worldwide.

Stem cells can be classified according to their origin. Their provenance also specifies their biological competence. As such, stem cells can be derived from embryonic or adult tissues and are thus termed embryonic stem cells (ESCs) or somatic stem cells (SSCs), respectively. With regard to biological competence, ESCs can differentiate and contribute to any of the three germ layers (ectoderm, mesoderm and endoderm) and are therefore pluripotent [1], whereas somatic stem cells are limited to differentiating into tissue-specific cell types [2]. Somatic stem cells can be classified as multipotent or unipotent depending on their differentiation capacity. While multipotent SSCs can give rise to multiple differentiated tissue-specific cell types, unipotent SSCs have a much more restricted potential and can only differentiate towards one cell type. 

Self-renewing mouse pluripotent ESCs (mESCs) were first derived from the inner cell mass (ICM) of pre-implantation blastocysts in the early 1980s [1,3]. The establishment of their culture and maintenance in vitro provided the basis for one of the best available models of mammalian early embryonic development. Embryonic stem cells enable the study of early cell fate decisions and the pluripotent state that characterizes them during the short timeframe from fertilization until the spatially-defined differentiation into multiple cell lineages and tissues that build the organism. For this reason, ESCs enable us to look at the origin of birth defects and understand how these could be prevented or perhaps reversed.

The ability to proliferate without limit and to differentiate towards any cell type of the body render ESCs as a limitless supply of specific cell types for basic research and transplantation therapies for many conditions, ranging from heart disease to neurodegenerative disorders. Furthermore, ESCs harbor an important potential not only for basic and translational research, but also for the improvement of pre-clinical safety and efficacy testing of novel pharmaceutical drugs.

Despite the vast and wondrous application potential of human ESCs (hESCs), their use remains very controversial due to their embryonic provenance. Moreover, histocompatibility issues are a major hindrance to the application of hESCs in regenerative medicine and cell transplantation therapies [4].

In 2006, a ground-breaking study by Shinya Yamanaka demonstrated that somatic cells can be reprogrammed to a pluripotent “ESC-like” state by overexpressing the pluripotency-inducing transcription factors OCT4, SOX2, c-MYC and KLF4. As a result, induced pluripotent stem cells (iPSCs) were born [5]. One great advantage of this new pluripotent cell category is that they permit autologous transplantation since obtaining material for reprogramming is as simple as collecting peripheral blood from the recipient, circumventing the issue of histocompatibility. Moreover, iPSCs capture the genetic landscape of the donor and are thus invaluable tools for modeling rare genetic disorders in vitro.

Altogether, the potential and impact of ESCs and iPSCs in basic and translational/clinical research are considerable. The possibilities for their application have been increasing steadily, indicating their full potential is yet to be known. Nevertheless, our understanding of the key pathways that rule self-renewal and differentiation of pluripotent stem cells remains far from complete. The signaling pathways and transcription factors that control early developmental stages are central in the regulation of the pluripotent state of ESCs and iPSCs. Therefore, it is not surprising that key features of the pluripotent state are governed by pathways such as leukemia inhibitory factor (LIF), sonic hedgehog, fibroblast growth factor (FGF), Nodal/ bone morphogenetic protein (BMP) and Wnt [6]. Indeed, all of them have been implicated either in the maintenance of self-renewal of pluripotent cells or in their differentiation capacity.

In this review, we will focus on the biological functions of the canonical Wnt pathway during early mammalian development, as well as its indispensable function on the regulation of ESC self-renewal, cell cycle and somatic cell reprogramming.

## 2. The Wnt Pathway

Wnt signaling is an evolutionarily-conserved pathway, which regulates multiple cellular processes including proliferation, cell polarity, stem cell self-renewal, differentiation and organogenesis during both embryogenesis and adult tissue homeostasis [7]. Its deregulation can cause severe developmental defects and has been linked to multiple pathological processes such as congenital and metabolic disorders and cancer [8,9]. Wnt signaling also has a key role in somatic cell reprogramming, which we will further discuss in this review.

The Wnt pathway was first discovered in *Drosophila melanogaster*, and it was named Wingless (Wg) due to its role as an essential morphogen for wing development [10]. Later, the *int-1* proto-oncogene was described to be able to promote mammary tumor formation in mouse [11]. Further research showed that both belong to the same evolutionarily highly-conserved signaling network, and therefore, the combination of *Wg* and *int-1* led to the currently-used nomenclature: Wnt (Wingless-related MMTV integration site) [12]. 

Wnt signaling has been categorized into two major branches: the canonical and the non-canonical Wnt signaling pathways. The canonical Wnt pathway, which will be discussed in more detail in this review, comprises a series of subsequent events that lead to the stabilization and translocation of β-catenin into the nucleus (see below). Non-canonical Wnt signaling (planar cell polarity and the Wnt/calcium pathway) does not involve stabilization of β-catenin, but requires Wnt ligands [13].

Wnt ligands are secreted glycoproteins produced by different cell types, which are thought to act in a mostly paracrine fashion [14,15]. In mammals, the Wnt family of ligands consists of 19 different members, which are cysteine-rich proteins containing one N-terminal signal peptide for secretion. Porcupine is an endoplasmic reticulum *O*-acyltransferase, which adds one palmitoyl group to Wnt proteins. This lipid-modifying step is critical for the extracellular secretion of Wnt ligands and their biological function [16,17]. Another important element in the Wnt secretory mechanism is the transmembrane protein Wntless, which facilitates the shuttling of Wnt ligands to the plasma membrane for secretion [18].

At the recipient cell, Wnt ligands bind to the seven-pass transmembrane receptor called Frizzled (FZ or FZD) and the co-receptors low-density lipoprotein receptor-related protein (LRP) 5 and 6, which are essential for signal transmission [19]. The interaction of Wnt ligands with the receptor complex can be modulated by secreted factors such as members of the Dickkopf (DKK) family, which prevent Wnt binding to the LRP receptors [20,21].

**Figure 1:**
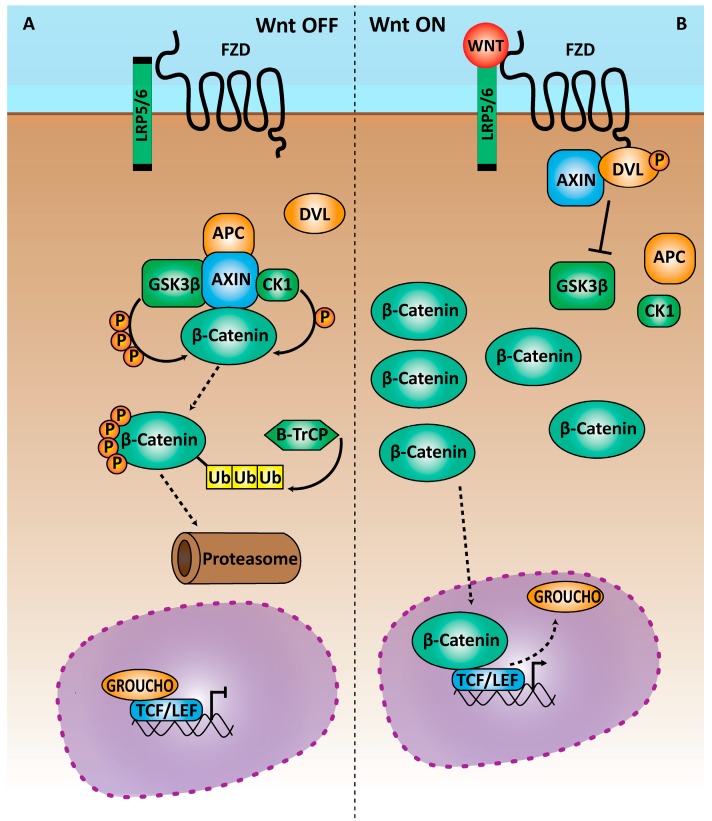
Molecular overview of the Wnt/β-catenin pathway. (**A**) In absence of Wnt ligands (Wnt OFF conditions), the β-catenin destruction complex phosphorylates β-catenin, which is ubiquitinated by β-TrCP and sent to the proteasome, where it is degraded. Thus, absence of nuclear β-catenin enables repression of Wnt target genes through the T-cell factor (TCF)/lymphoid enhancer factor (LEF) transcription factors. (**B**) When Wnt ligands bind to the receptor complex, the destruction complex is disassembled allowing the stabilization of β-catenin, which is then able to translocate to the nucleus. Nuclear β-catenin is then able to elicit gene expression changes through the TCF/LEF family of transcription factors. APC: adenomatous polyposis coli; CK1: casein kinase 1; DVL: dishevelled; FZD: frizzled; GSK3β: glycogen synthase kinase 3 beta; LRP5/6: lipoprotein receptor-related protein 5/6; Ub: ubiquitin.

In the absence of Wnt (Figure 1A), cytoplasmic β-catenin, the central molecule of the Wnt signaling pathway, is constantly degraded by the action of the destruction complex. This complex is composed of the scaffolding protein AXIN, the tumor suppressor adenomatous polyposis coli gene product (APC), casein kinase 1 (CK1) and glycogen synthase kinase 3 (GSK3β). While bound to AXIN, β-catenin is constitutively and sequentially phosphorylated in its amino-terminal region by CKand GSK3β [22]. When phosphorylated, β-catenin can be recognized by β-TrCP and the E3 ubiquitin ligase subunit and therefore targeted for ubiquitination and subsequent degradation. The continuous degradation of cytoplasmic β-catenin prevents its translocation to the nucleus.

When Wnt ligands bind to the receptor complex and initiate intracellular propagation of their signal (Figure 1B), GSK3β is inhibited, and β-catenin is stabilized. Consequently, the latter accumulates in the cytoplasm and translocates to the nucleus, where it activates the transcription regulation activity of T-cell (TCF)/lymphoid enhancer factor (LEF), driving the expression of many important genes for different cellular functions. The TCF/LEF proteins belong to a family of transcription factors, which include TCF1 (gene name *Tcf7*), LEF1, TCF3 (*Tcf7l1*) and TCF4 (*Tcf7l2*). TCF1, LEF1 and TCF4 can bind β-catenin and activate transcription of target genes as a result of Wnt signaling. In contrast, when the Wnt pathway is not active, all the TCF/LEF factors can recruit repressive complexes and function as repressors of transcription of many target genes [23]. 

Wnt targets include genes responsible for regulating cell proliferation, stem cell homeostasis and important developmental processes. A list of the many target genes of the Wnt pathway can be found on the web page “The Wnt Homepage” of the Nusse Laboratory [24].

## 3. Activity and Role of the Wnt/β-Catenin Pathway during Mammalian Pre-Implantation Development

Mammalian embryogenesis is regulated by a crosstalk of several key signaling pathways that govern the correct development of the embryo. Murine models provided numerous important insights in this field. In addition, during the last 30 years, mouse ESCs have been an invaluable in vitro tool to probe the function of genes involved in early embryogenesis and to study the signaling networks and gene expression landscape governing these first stages of development.

Among these pathways, Wnt/β-catenin has been described to play an important role in different stages of embryogenesis. For instance, during gastrulation, Wnt signaling is the force that drives the acquisition of the primitive streak cell fate, establishing the anterior–posterior axis of the mammalian embryo [25,26]. 

**Figure 2:**
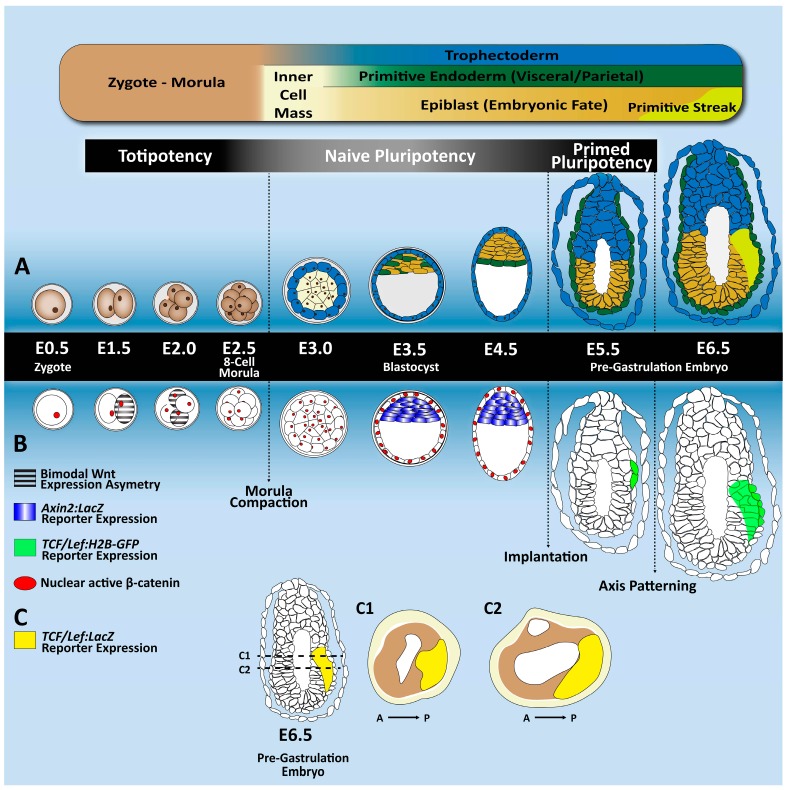
Tracing Wnt activity across early mouse embryogenesis. (**A**) A schematic overview of the mouse pre-implantation and post-implantation developmental stages, from zygote formation (E0.5) until the pre-gastrulation stage (E6.5). After fertilization, the zygote undergoes a series of mitotic divisions together with progressive cell fate acquisition. At the end of the morula stage, the first segregation event occurs giving rise to the trophectoderm and the inner cell mass (ICM). At E4.5–E5.0, after the ICM segregates into the epiblast and primitive endoderm, the blastocyst implants in the uterus. Around E6.5, the egg cylinder is formed, and anterior–posterior axis patterning is established, along with the first mesendodermal progenitors at the primitive streak. (**B**) This chart provides information about the main molecular changes in the Wnt/β-catenin signaling pathway. The bimodal Wnt target gene expression is represented in grey stripes and is (at transcript level) already detected at the two- and four-cell stages [27]. In red, active nuclear β-catenin expression [28]. In blue, the *Axin2*:*LacZ* reporter is found only at the blastocyst stage [29]. In green, detection of the TCF/Lef:Histone 2B-green fluorescent protein (H2B-GFP) reporter occurs only after implantation stages [30]. (**C**) Longitudinal and transversal sections of a pre-gastrulating mouse embryo (E6.5) showing in yellow the distribution of the *TCF/Lef:β-galactosidase* reporter activity in the posterior region [30].

The role of the Wnt signaling pathway in the earliest stages of mammalian development remains largely unknown and somewhat controversial. In this section, we will attempt to summarize what is currently known regarding the role of key components of the canonical Wnt signaling pathway from the very beginning of embryogenesis until the implantation blastocyst stage (Figure 2).

As mentioned above, *Mus musculus* has long been the most important tool for the study of mammalian embryonic development, and this review will focus on this model, drawing parallels with embryonic development of humans whenever possible.

Components of the Wnt signaling pathway can be detected at RNA level during the first stages of embryonic development, suggesting it may have a functional role during the earliest meanders of embryogenesis. Nonetheless, whether Wnt signaling is essential is still a controversial topic. Therefore, intensive research has been performed during recent years in order to validate the functions and importance of the Wnt pathway during embryogenesis and embryonic development at protein and functional levels (Figure 2A–C).

### 3.1. From Zygote to Late Morula Stage (E0.5–E2.75)

Upon fertilization, the mouse zygote (one-cell stage) undergoes a succession of cleavages (cell division without cell-growth), giving rise to a mass of cells named the morula. At this point, the zygote is transcriptionally silent and inactive, and maternal mRNAs and proteins are tasked with initiating and controlling the first stages of embryonic development [31]. Different Wnt ligands, receptors and related regulators have been detected at transcript level at this stage [31]. Finally, the mouse embryo exits this period of transcriptional silence at the two-cell stage, when embryonic genome activation (EGA) occurs. 

Embryonic genome activation is a potential source of transcriptome asymmetry in each of the blastomeres, both at the two and four-cell stages. It has been theorized that the manifest bimodal gene expression of Wnt receptors and Wnt-related transcription factors in one of the blastomeres, but not in the other, is governing this process during mouse embryogenesis [27]. 

Wnt ligands (*Wnt3a*, *Wnt5a*, *Wnt7a*) [32] and most of the canonical Wnt receptors (*Lrp5–6* and *Fzd1–9*) [31] have been detected at the transcript level as early as during the transition between the zygote and two-cell stage of mouse embryogenesis. Additionally, RNA-sequencing has recently enabled the detection of different Wnt component transcripts (*Axin2*, *Lef1* and *Lrp5–6*) during the four to eight-cell stage (E1.5). These are highly expressed when compared with the ensuing stages of embryonic development [33]. 

Active nuclear β-catenin can be detected as early as from the zygote until the late morula [28] highlighting a possible role of canonical Wnt signaling during the first stages of embryonic development. This same study also reports that inactivating Wnt signaling from the two-cell stage onwards by overexpressing the canonical antagonist DKK1 ultimately renders the blastocyst incapable of implanting in the uterus [28]. 

Nevertheless, several laboratories have reported that β-catenin null mutant mouse embryos develop normally until the blastocyst stage. Moreover, both *Wnt3a* null and double *Tcf1/Lef1* null embryos develop properly until gastrulation (E6.5–E7.0), at which point they fail to build the paraxial mesoderm [34]. In line with these results, β-catenin (*Ctnnb1*) null embryos implant normally only showing developmental problems also at the gastrulation stage, where abnormalities in the establishment of the anterior–posterior axis and mesoderm specification occur [35,36]. However, in these experiments, it is not possible to rule out that maternally derived β-catenin could have a rescuing effect, thereby allowing embryos to develop until the E6.5 stage. To rule out this possibility, De Vries et al. depleted both maternal and zygotic β-catenin in oocytes, observing that the embryos can develop ex vivo, reaching the blastocyst stage even though the number of pups born from females harboring a partial deletion of β-catenin was much lower compared to control females [37].

The absence of a clear phenotype in early β-catenin null embryos is surprising since the components and target genes of the pathway are already expressed in the zygote and at the morula stage. This could be explained by the presence of other proteins, which may have redundant functions with β-catenin, thus rescuing the outcomes of its absence in null embryos. In fact, other members of the catenin family have shown a certain level of redundancy with β-catenin. Plakoglobin (γ-catenin) has been demonstrated to rescue the cell-adhesion functions of β-catenin in mESCs lacking expression of the latter [38]. Importantly, plakoglobin is expressed from the eight-cell stage onwards in the mouse embryo [39], raising the possibility of the existence of a compensatory mechanism in place to suppress the outcomes of β-catenin depletion during embryogenesis. 

Whether other signaling pathways could compensate the disruption of Wnt/β-catenin signaling during mouse embryonic development needs to be further explored before concise conclusions can be drawn. Nonetheless, other signaling pathways seem to appear in the same conflicting pattern as β-catenin during early embryonic development, namely the leukemia inhibitor factor/signal transducer and activator of transcription (LIF/STAT3) pathway. 

Similarly to Wnt, LIF signaling plays a crucial role in maintaining pluripotency both in vivo (in the ICM) and in vitro (in mESCs) [40]. From the four-cell stage until the blastocyst, STAT3 transcriptional activity is dependent on the LIF growth factor. Much like in β-catenin null embryos, depletion of STAT3 does not impair the correct development of the morula, but proves to have a critically negative impact on the ability of the blastocyst to survive and implant [41]. The critical role of LIF and Wnt in the maintenance of pluripotency, but the absence of developmental defects when one or the other is perturbed suggest a possible reciprocal rescue capacity of one pathway upon the other during early embryonic development.

### 3.2. From Late Morula to Blastocyst Implantation Stage (E2.75–E4.5)

Successive cell divisions at the late morula stage lead to compaction of the embryo and the rise of the first cell lineages in the blastocyst: (i) the trophoblast, which will go on to make up the placenta, and (ii) the ICM, which will later segregate into the epiblast and the primitive endoderm [42].

Implantation of the mouse blastocyst occurs during E4.5–E5.0 (Figure 2), constituting a critical step of embryonic development and requiring a fine-tuned synchronization of blastocyst activation and uterine receptivity [43]. Critically calibrated modulation of the Wnt signaling pathway is fundamental during this process. In fact, forced activation of canonical Wnt signaling in ex vivo pre-implantation embryos prevents progression towards the implantation stage and hinders the correct hatching of both mouse [44] and bovine embryos [45]. This suggests that calibrated modulation of the Wnt pathway is crucial for the final development of the blastocyst. Components of the Wnt signaling pathway follow a specific expression pattern in the embryo and in the uterus, both during pre-implantation development and at the implantation stage. For instance, transcripts of canonical Wnt ligands *Wnt3a*, *Wnt6*, *Wnt7b*, *Wnt10b* and *Wnt1* is clearly detected in blastocysts [46,47]. Moreover, canonical members of the *Fzd* and *Lrp* receptor families are also expressed during this developmental stage.

Even though activity of the β-catenin activated transgene-β-galactosidase (BAT:gal) or the TCF/Lef:histone2B-green fluorescent protein (*TCF/Lef:H2B-GFP*) reporters have not been detected in mouse embryos during both the pre-implantation and implantation stages [30,48], expression of the specific Wnt target *Axin2* has been confirmed in the ICM during early and late blastocyst stages, using an *Axin2*:*LacZ* reporter [29]. Moreover, elevated levels of active β-catenin [48] can be detected in murine embryos during the implantation stage.

Altogether, these data suggest an important role of the Wnt pathway in the ICM during the pre-implantation and implantation stages, which is in accordance with its role in maintaining the pluripotency of ESCs (see below). 

However, Xie et al. have reported that expression of WNT3A and the presence of active nuclear β-catenin are maintained in the trophectoderm, but not in the ICM during the peri-implantation stage (E4.5–E5.0) [28]. A possible interaction between the Wnt and Hippo signaling pathways could underlie this phenomenon. The latter is known to be involved in the specification of the trophectoderm vs. ICM fates. In summary, when the Hippo pathway is inactive, Yes-associated protein/transcriptional coactivator with PDZ-binding motif YAP/TAZ becomes stabilized and accumulates in the nucleus where it binds Tead4 to drive expression of genes involved in trophectoderm induction, such as *Cdx2* [49]. Interestingly, one study in *Drosophila melanogaster* describes that when YAP/TAZ is absent in the cytoplasm, Dishevelled (DVL) is easily phosphorylated, ultimately driving the disruption of the β-catenin destruction complex and in turn switching on the Wnt pathway [50]. As such, this potential crosstalk between the Wnt and Hippo pathways could explain the presence of both WNT3A and nuclear β-catenin in the trophectoderm. Therefore, a more detailed study of the interception points of different signaling pathways involved in embryogenesis and development is required in order to establish a definitive causality of early cell fate decisions.

In human blastocysts, WNT3 and membrane-associated β-catenin specifically accumulate in the trophectoderm cell layer at the blastocyst stage [51]. Furthermore, degradation of β-catenin impairs the formation of the trophectoderm underlining the importance of the Wnt/β-catenin pathway in the regulation, albeit not transcriptionally, of the first cell fate decision in human embryonic development [51].

The timely shutdown of Wnt/β-catenin signals is just as important as the stimulation of the pathway in the context of embryonic development. One such case seems to be the specification of the primitive endoderm (PrEn) in mouse embryos. Boroviak et al. sequenced the transcriptome of single embryos to demonstrate that the antagonist of the canonical Wnt pathway *Dkk1* is specifically expressed in the cells that segregate from the ICM to give rise to the PrEn at E4.5 [33]. Moreover, to this day and the best extent of our knowledge, no signs of activation of the Wnt pathway in the primitive endoderm have been detected at this stage of development. 

Although several studies in mice pinpoint the importance of the FGF/Extracellular-signal-regulated-kinase (ERK) pathway in the determination of the epiblast vs. primitive endoderm cell fate decision [52,53], this function is not conserved in human embryos [54]. Therefore, further research is required to understand which other signal pathways (among them, Wnt) might be regulating PrEn specification in the human model.

In conclusion, Wnt/β-catenin signaling fluctuates during the first developmental stages, and it is difficult to assess and determine its activity. In mice, although its nuclear transcriptional activity is dispensable for the development until blastocyst stage, its absence impairs blastocyst competency and implantation in the uterus. Expanding the knowledge of the crosstalks and interactions of different pathways involved in the earliest stages of development should be given attention in future research efforts. For instance, it would be important to understand whether depletion of STAT3 and β-catenin influences the process of morula compaction or whether one pathway could compensate the absence of the other to support correct embryonic development.

## 4. The Role of the Wnt/β-Catenin Pathway in the Regulation of Naive and Primed Pluripotency of Mouse and Human Embryonic Stem Cells

Embryonic stem cells boast two remarkable characteristics: pluripotency and self-renewal. Pluripotency is a dynamic cell state that occurs naturally during a very limited and narrow window of time of embryonic development. During pre- and early post-implantation of the blastocyst, cells of the ICM of the developing embryo have the ability to commit to any of the three germ layers (ectoderm, endoderm and mesoderm), which will eventually give rise to all the specialized cell types that constitute the adult organism [6]. In addition, ESCs are able to self-renew, giving rise to at least one pluripotent stem cell with each division.

Depending on the developmental stage of the embryo from which ESCs are derived, we can distinguish two types of pluripotency: naive and primed pluripotency. Naive mouse ESCs are derived from the epiblast of pre-implantation blastocysts and preserve ICM-like molecular features in vitro. On the other hand, primed ESCs are isolated from the epiblast of post-implantation embryos, displaying a completely distinct epigenetic and transcriptional landscape when compared to naive ESCs [6,55].

Naive mESCs were the first pluripotent cells to be established in culture [1,3]. These cells are the gold-standard in vitro tool to study the molecular mechanisms regulating mammalian pluripotency and differentiation. Growth conditions are crucial to maintain pluripotency, which ultimately relies on the artificial in vitro self-renewal state generated by supplementing ESCs with appropriate cytokines and growth factors. Initially, ESCs were co-cultured with mitotically inactivated fibroblasts (MEFs) in fetal bovine serum (FBS)-enriched conditions [1]. However, both growth factor secretion by MEFs (the “ingredients” of pluripotency), the presence of serum (FBS) and consequent batch-to-batch variability introduce inconsistency in culture conditions. Providentially, LIF, an activator of the Janus kinase (JAK)/STAT pathway, was recognized to vastly improve culture conditions by enabling the propagation of ESCs without the necessity of MEFs in the presence of serum [56,57]. 

**Figure 3:**
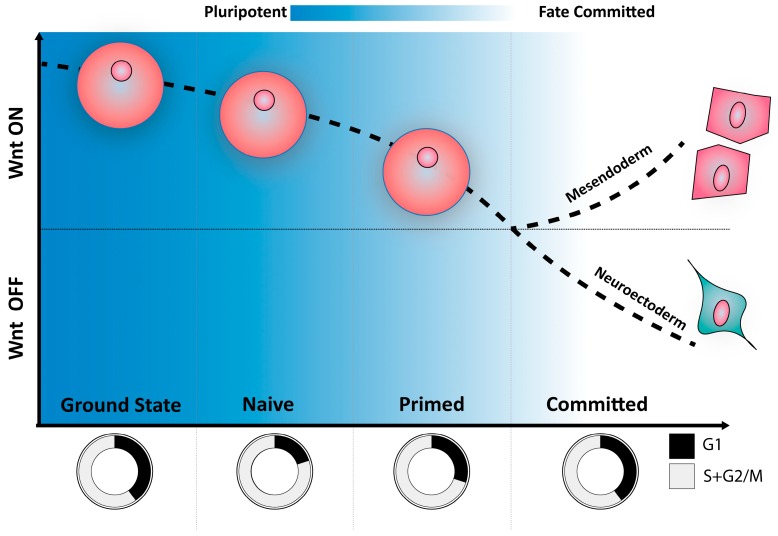
Wnt/β-catenin modulation correlates with different pluripotency and cell cycle stages. In mouse embryonic stem cells (mESCs), activation of the Wnt signaling pathway maintains naive pluripotency. Naive pluripotent conditions with Wnt induction or ERK inhibition correlate with a slow-down in cell cycle progression. Once the naive state is maintained without these inhibitors (i.e., serum + Leukemia Inhibitor Factor (LIF) conditions), mESCs start to proliferate faster. The inhibition of Wnt induces the cells to differentiate towards a primed pluripotency state (epiblast stem cells (EpiSCs)). For further differentiation commitment, activation is crucial to induce mesendoderm cell fate, while an acute repression of the pathway will generate neuroectoderm cells. In addition, cells differentiating show a classic slow cell cycle.

Before these achievements, Wnt signaling had not yet been considered as a possible regulator of pluripotency and self-renewal of mESCs. In 2004, Sato et al. demonstrated for the first time that activation of Wnt signaling through inhibition of GSK3β, a component of the β-catenin destruction complex, was able to maintain mouse pluripotency in absence of LIF [58]. In agreement, the addition of repressors of Wnt-secretion to FBS/LIF medium induces loss of pluripotency and differentiation towards a primed epiblast stem cell state [29] (Figure 3). The presence of *Axin2:LacZ* reporter activity in the ICM (E3.5) [29], the developmental stage from which mESCs are derived, backs a potential role of canonical Wnt signaling in the maintenance naive pluripotency.

All these culture conditions generate an intraculture heterogeneity in pluripotency due to the presence of serum. A key step towards the establishment of a homogenous pluripotent state free from extrinsic stimuli was the achievement of the naive ground state of pluripotency through the use of the defined 2 inhibitors (2i) small molecule combination, which consists of MEK inhibition together with Wnt stimulation (through GSK3β inhibition) [59]. Furthermore, GSK3 and MEK inhibitors together with LIF (2iLIF) enhance the expansion and growth of mESCs. 

Over the years, it has been shown that mESCs maintained in 2iLIF conditions (or ground state) express homogenous transcript levels of components of the pluripotent transcriptional network and are less prone to differentiate. Moreover, mESCs in the ground state contribute more efficiently to chimera formation in comparison to cells grown in serum [60,61]. The 2i culture setting homogenizes the transcriptional and epigenetic landscape of mESCs, allowing a robust and replicable starting point for many differentiation protocols. Notwithstanding this, homogenization buries interesting aspects and cell types, which arise in serum-grown cells, namely primed mESCs [62], PrEn progenitors [63] and totipotent cells (capable of generating both embryonic and extraembryonic tissues) [64].

Surprisingly, upon withdrawal of LIF, the stabilization of β-catenin and inhibition of ERK can bypass the otherwise statutory LIF/STAT3 signaling to maintain self-renewal and naive pluripotency [59]. In fact, the combination of only two out of these three pathways (Wnt activation, LIF activation or ERK inhibition) sustains pluripotency and self-renewal, alluding to a potentially redundant and/or compensatory effect between them. In fact, mESCs maintained in the presence of FBS and Wnt3a conditioned media or GSK3β inhibitors are able to preserve self-renewal and the core pluripotency network without requiring LIF [58]. Indeed, it has previously been demonstrated that activation of Wnt/β-catenin upregulates the expression of STAT3, aiding to the maintenance of pluripotent mESCs and demonstrating a synergistic effect between the LIF pathway and Wnt pathways [65]. Recent research points to a synergy of both pathways to stabilize pluripotency through the *Sp5* gene in mESCs [66]. Therefore, we can conclude that pluripotency is not regulated by a discrete signaling pathway. Instead, there is a combination of distinct pathways that perform redundant functions towards its maintenance. Among them, the Wnt canonical pathway and specifically β-catenin stabilization have emerged as some of the major characters in this process.

Herein, we will expose the molecular evidence underlying the role of Wnt/β-catenin in self-renewal and pluripotency maintenance, as well as the controversy regarding whether it is or not indeed an absolute requirement. 

In mESCs, depletion of both GSK3 isoforms (GSK3α/GSK3β) or mutation of the two *Apc* alleles, both components of the β-catenin destruction complex, results in an increase of β-catenin stabilization and hinders the differentiation of mESCs in teratomas or embryoid body assays [67,68]. Concretely, double GSK3 depletion results in aberrant TCF/LEF activity and constitutive expression of Wnt targets (*Axin2*, *Sp5*, *Cdx-1*) in mESCs maintained in Wnt depleted medium, confirming that GSK3β inhibition stabilizes pluripotency through Wnt/β-catenin, and not by off-target effects [68]. Furthermore, the overexpression of a stabilized isoform of active β-catenin was able to induce self-renewal, abrogating differentiation in mESCs maintained in the absence of LIF [69,70]. Moreover, Wnt ligands have been shown to induce polarization and asymmetric division of mESCs. Specifically, when exposed to immobilized Wnt ligands, mESCs give rise to daughter cells with distinct fates, wherein the cell lying proximal to the ligand retains self-renewal and pluripotency, but not its sister, distal to the Wnt ligand, which exits pluripotency and differentiates [71].

However, similar to the observations of early development studies, the importance of the Wnt pathway in maintaining and establishing pluripotency faces another important paradox: despite creating instability in the adhesion and growth capacities of mESCs, the complete ablation of β-catenin does not impair self-renewal. Furthermore, these cells retain expression of pluripotency markers both in FBS/LIF [38] or 2iLIF culture conditions [72]. This could be explained by possible compensating roles of LIF and the Wnt pathway on pluripotency maintenance. In fact, Smith and colleagues reported that depletion of β-catenin inhibited self-renewal in 2i conditions, but not in 2iLIF, highlighting the fact that GSK3β inhibition and LIF have complementary roles and that β-catenin protein stabilization is important, albeit not essential, for pluripotent self-renewal [72].

Recent studies have emphasized the importance of the TCF/LEF family of transcription factors. Mouse ESCs express all four TCF/LEF members, although the single or quadruple knock-out does not impair pluripotency, supporting the idea that its transcriptional regulation is independent of Wnt signaling, even in the absence of LIF [73]. However, it has been shown that β-catenin does not exclusively elicit regulation of gene expression through TCF/LEF mediation. In fact, it can reinforce pluripotency, even in the absence of LIF, by interacting with the transcription factor OCT4 [74]. Thus, a TCF/LEF independent role of β-catenin may support an alternative pluripotency maintenance mechanism.

Among all the TCF/LEF factors, TCF7L1 (also known as TCF3) is the most expressed in mESCs. Its genetic ablation mimics Wnt signaling induction, since *Tcf3* null mESCs maintain self-renewal [75]. Moreover, *Tcf3* deletion delays differentiation of mESCs when propagated as embryoid bodies (EBs) (3D culture) [72,75,76]. 

Chromatin immunoprecipitation and sequencing analysis (ChIP-seq) of TCF3 had a considerable impact on our understanding of its functions [77]. These experiments demonstrated that the DNA binding sites of TCF3 overlap more than 70% with those of the pluripotent factors OCT4, NANOG and SOX2, and therefore, TCF3 regulates transcription of the same genes. Surprisingly, while knockdown of *Nanog* and *Oct4* in mESCs disrupts pluripotency, *Tcf3* null mESCs maintain or even increase it [78]. Moreover, knockdown of *Nanog* in *Tcf3* null mESCs does not impair pluripotency, suggesting an independent role of TCF3 in the core pluripotency network [75]. Different ChIP-seq studies confirmed that TCF3 binds to the enhancer regions of these two pluripotent transcription factors and together with experiments making use of the *TOPGFP* reporter (which contains seven Wnt response elements to drive the expression of green fluorescent protein) suggested that TCF3 directly or indirectly executes a repressor activity on the maintenance of pluripotency and self-renewal [76,79]. Furthermore, TCF3 overexpression corroborated that it indeed represses the stem cell pluripotency program [80]. In addition, a peculiar and vital target of TCF3 repression is the *Esrrb* gene. ESRRB expression in mESCs is sufficient to mediate the pluripotency-inducing effects of GSK3β inhibitors [81]. In 2i conditions, removal of GSK3β inhibitors does not impair pluripotency when *Esrrb* is ectopically expressed [81]. Altogether, TCF3 exerts a suppressive function on self-renewal of mESCs and is part of the core pluripotency network.

### The Pro-Differentiating Role of the Wnt Pathway in the Primed Pluripotent State

Aggregated mESCs can generate early embryo-like structures named EBs, which follow a roadmap of embryonic lineage specification similar to that of embryonic development. As such, embryoid bodies are extremely useful tools to study lineage commitment and tissue formation. It has been observed that Wnt signaling is able to polarize EBs giving rise to a primitive streak-like region that co-expresses mesendodermal markers, advocating a pro-differentiation role of Wnt pathway [82]. In addition, primed mouse pluripotent cells (epiblast stem cells (EpiSCs)) undergo mesendoderm differentiation upon Wnt stimulation, escaping from the pluripotent state [83]. Therefore, Wnt signaling has opposite cellular functions depending on the pluripotent cell state: while its activation promotes maintenance of pluripotency in the naive state [58], it drives differentiation of primed pluripotent cells [83].

Human embryonic stem cells were first isolated from pre-implantation blastocysts [84] in 1998 and much like mESCs have the ability to differentiate towards the three embryonic lineages and self-renewal capacity. However, the currently available conditions for in vitro culture of hESCs, which include the modulation of FGF2 and transforming growth factor-β1 (TGFβ1)/Activin A [85], render a transcriptional and epigenetic signature, which resembles more closely that of mouse EpiSCs. In other words, hESCs are closer to post-implantation epiblast and retain a large number of features from primed pluripotency. These include low levels of expression of naive pluripotency markers (such as *DPPA3*, *KLF2* and *ESRRB*), deposition of H3K27me3 over developmental genes, absence of global DNA hypomethylation, lack of activity of the *OCT4* distant enhancer, absence of a pre-X-chromosome inactivation state in most female hESC cell lines and very low chimeric potential [86]. Moreover, in accordance with what happens in primed mESCs, activation of the Wnt pathway induces mesendodermal differentiation in hESCs [87,88,89].

Naive and primed metastable potency states can be attributed to determined cell signals present in in vitro culture conditions as shown by the possibility to convert mouse PSCs into primed or naive pluripotent states by modifying culture conditions (see above). However, in contrast with mESCs, 2i/LIF culture conditions are not sufficient to maintain naive human ES cells or iPSCs. Nonetheless, during the last five years, several groups have attempted to achieve the in vitro naive pluripotent state in hESCs [90,91,92,93,94]. The strategies followed differ between groups, but are mainly based on the use of small molecules and growth factors to modulate important signal pathways, which balance pluripotency and differentiation, such as Wnt, ERK1/2, LIF, BMP, JNK, p38 or protein kinase C (PKC). In addition, overexpression of transcription factors such as KLF2 and NANOG, combined with some of the aforementioned molecules induces the naive human pluripotent state [91]. In all cases, in agreement with the role of the Wnt pathway on the maintenance of mouse naive pluripotency, a GSK3β inhibitor has been used to promote the human naive state.

Human ESCs generated in all these conditions show higher levels of expression of naive pluripotency markers (such as *DPPA3*, *KLF2* and *ESRRB*), release of histone methylation marks such as H3K27me3 over developmental genes, presence of global DNA hypomethylation as seen in ICM cells, *OCT4* proximal enhancer activity and a pre-X-chromosome inactivation state in most female hPSC lines [86]. Furthermore, Yang and colleagues have recently demonstrated that their naive pluripotent state conditions allowed human PSCs to contribute both towards embryonic and extraembryonic tissues (extra-embryonic endoderm and placenta) with a high grade of inter-species chimerism [94].

## 5. Regulation of Pluripotent Stem Cell Cycle and Proliferation by the Wnt Pathway

The tumorigenic potential of stem cells is one of the main issues holding back their widespread application in regenerative therapies. Stem cells have virtually infinite proliferation capacity and a uniquely fast cell cycle. Obtaining control of its intricacies is of great importance and would potentially increase the safety of the use of stem cells in the treatment of many important medical conditions of our time. Accordingly, interest in the subject has been increasing, and with every research effort, novel insight into the unique properties of the pluripotent cell cycle brings us closer to understanding the critical aspects of its regulation.

The pluripotent cell cycle differs from that of somatic cells. It stands at the center of two key and indivisible processes of stem cell biology: proliferation and self-renewal. As such, the cell cycle is widely considered to play a key role in the maintenance of balance between stemness and differentiation signals (Figure 3). 

When cultured in traditional serum LIF conditions, mouse embryonic stem cells have an unusually short cell cycle characterized by very short G1 and G2 phases, coupled with a longer than usual S phase resulting in a doubling time of 12–14 h [95]. This can be attributed to the lack of expression of cell cycle regulator proteins cyclin-dependent kinase inhibitors (CDKIs) belonging to the *Cip/Kip* and *Ink/Arf* families and the presence of constant CyclinE/CDK2 activity regardless of the cell cycle stage [96]. Constitutive CyclinE/CDK2 induces a bypass of the early G1 phase by omitting retinoblastoma(RB)-mediated control of early G1 to late G1 transition [95]. Moreover, embryonic stem cells in the naive state lack competent DNA damage-induced p53/p21 cell cycle control [97] and present dynamic transcription of *E2f* target gene [98].

The G1 phase of the pluripotent cell cycle is regarded as pivotal in the regulation of cell fate decisions, being frequently described as the moment during which stem cells are most prone to differentiation cues. By fluorescence-activated cell sorting (FACS) sorting mESCs in different cell cycle stages using the fluorescence ubiquitination cell cycle indicator (FUCCI), Coronado et al. demonstrated that stem cells in the G1 phase are more susceptible to differentiation cues. Moreover, Coronado demonstrated that transition from the naive pluripotent state towards the less potent epiblast-resembling primed state is accompanied by a lengthening of the G1 phase (Figure 3) [99].

As such, the short G1 phase has been thought to serve as a pluripotency protective mechanism, which narrows the time window during which ES cells can be coerced to differentiate. Additionally, a significant lengthening of the cell cycle accompanies differentiation. Specifically, the G1 phase becomes enlarged as the once pluripotent cells come under regulation of the p53 and Rb-dependent cell cycle checkpoints. 

Recently, it has been demonstrated that stem cells show a certain level of specificity as to which differentiation cues are permitted in the early or late G1 phase. Specifically, human ESCs seem to only commit to neuroectoderm differentiation while in the late G1 phase, whereas commitment towards the endoderm cell fate is permitted during the early G1 phase [100]. This differential capacity to perceive differentiation signals at specific cell cycle stages is controlled by the activity of cell cycle regulators. Regarding neuroectoderm specification, its chronological restriction to the late G1 phase is due to Cyclin D/CDK4-6-mediated inhibition of Activin/Nodal signaling. Endoderm differentiation, on the other hand, requires active Activin/Nodal signaling [100]. These findings highlight the tight control of the cell cycle over cell fate decision and bring to the spotlight the role of cell cycle regulators beyond the cell cycle itself.

Several recent studies have shown that in certain conditions, a lengthening of the cell cycle does not necessarily correlate with a loss of potency. Deletion of *c-Myc* in mouse embryonic stem cells maintained in 2i conditions has been shown to induce a phenotype resembling the naturally-occurring embryonic diapause, which can occur during development of several invertebrate and mammalian species, including mice, as a strategy to improve reproductive fitness. Importantly, c-MYC depletion-induced arrest can be rescued without loss of pluripotency by re-expression of exogenous c-MYC or by withdrawal of pharmacological inhibition of the transcription factor [101].

Interestingly, a very recent study from Huurne et al. may have induced a tectonic shift in our understanding of the regulation of the pluripotent cell cycle by challenging the notion that mESC lack typical G1 regulation, making use of bromodeoxyuridine and propidium iodide stainings and flow cytometric analysis to determine cell cycle distribution of mESC maintained in 2i and serum conditions. Their flow cytometry results point out important differences in the cell cycle profile of both culture conditions: in serum mESCs, the number of cells found in S phase is considerably higher than that of 2i mESCs, while the number of G1 phase cells is increased in 2i mESCs. Additionally, making use of the FUCCI reporter, Huurne et al. determined the G1 phase of 2i cells to be “markedly longer” than serum mESCs, along with a shorter S and G2 phase [102].

A short G1 phase may not be an inherent feature of stem cell biology, but rather a characteristic that arises from culture conditions and their similarity with different stages of embryonic development. Huurne further contributed to understanding the mechanisms of differential regulation of the cell cycle in 2i and serum conditions by demonstrating that ERK pathway activity induces a decrease in hypo-phosphorylated RB and loss of the G1 restriction point culminating in a condensation of the G1 phase. 

The Wnt signaling pathway is known to support maintenance of pluripotency and to be importantly involved in controlling somatic cell reprogramming [58,103]. It is a pro-mitotic pathway, and its deregulation is associated with the onset of many neoplastic disorders, but until recently, it was unclear whether it had a role in the pluripotent cell cycle. Recently, it was demonstrated that activation of the Wnt pathway has a double role in mESCs: it promotes expression of negative cell cycle regulators repressing cell cycle progression and proliferation while also reducing the expression of differentiation genes without disturbing pluripotency. Moreover, these effects are driven specifically through TCF1-dependent transcriptional regulation. In fact, a novel palindromic DNA binding motif through which TCF1, but not TCF3, regulates transcription of the *Ink4/Arf* tumor suppressor locus to drive expression of p16^Ink4a^ and p19^Arf^ has been described in the same study. Moreover, Wnt activity in mESCs was shown to result in increased nuclear p53 and depressed c-MYC expression [104]. These results are rather surprising considering Wnt’s “fame” as a proliferation-supporting pathway and its indispensable role in supporting proliferation of many somatic cell types by inducing expression of c-MYC. Moreover, this study highlights the cell type specificity of the outcomes of Wnt activation. In addition, they are in line with previous reports that agonists of the Wnt pathway severely hinder proliferation of the pluripotent cells of bovine blastocyst ICM [45].

Much is yet to be known about the pluripotent cell cycle and its functions. It is, however, clear that it is a highly dynamic cellular function, which, like an octopus, reaches far and wide to exert some level of control over each and every aspect of the pluripotent cell’s life. Wnt signaling seems to also have a dynamic role, and the outcomes of its activation may depend not only on the strength of its signal (self-renewal vs. dormancy), but also on the developmental/pluripotency state of the cell (differentiation vs. self-renewal) (Figure 3). Regardless of the outcome, Wnt signaling and the cell cycle seem to go hand-in-hand, and both have a first and last word on the pluripotent cell fate.

## 6. Wnt and Somatic Cell Reprogramming

The technology to induce murine and human somatic cells to revert to a pluripotent state (iPSCs) by way of transduction of several transcription factors (OCT3/4, SOX2, c-MYC, KLF4, NANOG and LIN28) has created the possibility of generating individualized pluripotent cells for patient-specific therapy or drug screening [5,105]. Reprogramming can also be accomplished by fusion of embryonic stem cells with somatic cells, thereby forming tetraploid cells in which the somatic genome induces the expression of pluripotent-associated genes and silences those associated with the somatic phenotype [106,107].

The current reprogramming methods remain very inefficient and yield a high number of cells that fail to undergo complete reprogramming [108,109]. Thus, several events that at present are not completely understood need to occur to correctly achieve somatic cell reprogramming to the pluripotent state. Understanding the mechanisms involved in self-renewal and reprogramming could lead to more efficient reprogramming strategies. In turn, this would ultimately enable large-scale production of fully-reprogrammed cells and the generation of patient-specific iPSCs for clinical application.

Growth factors and signaling pathways are the main external players in maintaining self-renewal and potency of pluripotent cells in vitro [6]. As described above, activation of the Wnt pathway is necessary and sufficient for the self-renewal of naive ESCs [29,58]. This could suggest an important role of canonical Wnt signaling in somatic cell reprogramming. In fact, in the original iPSC studies, β-catenin was found to promote reprogramming. However, it was discarded from the final cocktail of reprogramming factors (OCT4, SOX2, c-MYC, KLF4) [5], indicating that Wnt pathway activation is not a quintessential requirement for direct induction of somatic cell reprogramming.

**Figure 4:**
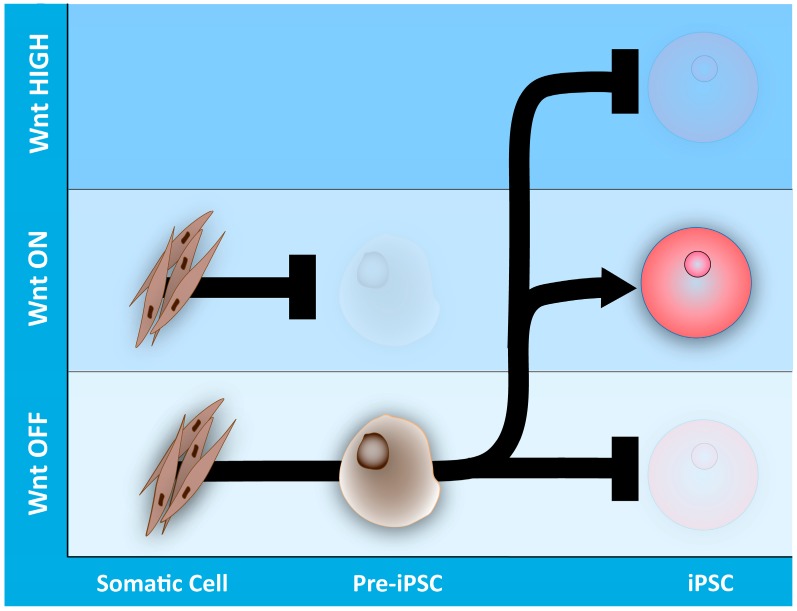
Temporal perturbation of the Wnt signaling pathway modulates somatic cell reprogramming efficiency. The levels of Wnt pathway activation during somatic cell reprogramming need to be precisely modulated in order to reach complete reprogramming. Somatic cells showing low Wnt activity (Wnt OFF state) at early stages of reprogramming and activation of the Wnt pathway (Wnt ON state) at late reprogramming stages, respectively, will result in completely reprogrammed induced pluripotent stem cells (iPSCs). In contrast, somatic cells with Wnt ON at early reprogramming stages or Wnt OFF at late stages produce partial or non-reprogrammed cells. It has also been demonstrated that high or aberrant levels of activation of the Wnt pathway have an inhibitory effect on somatic cell reprogramming.

Nevertheless, several recent studies have focused their attention on the central importance of the Wnt/β-catenin pathway as both an essential element to achieve reprogramming and on how its modulation affects the efficiency of the reprogramming process itself. Indeed, canonical Wnt signaling has been shown to be an important player in both fusion-mediated and factor-induced reprogramming in mouse and human somatic cells [103,110] (Figure 4). The use of either Wnt ligands (such as WNT3A), GSK3 inhibitors (CHIR99021 or 6-bromoindirubin-3’-oxime, BIO) or viral transduction to overexpress a stabilized form of β-catenin has beneficial effects on somatic cell reprogramming efficiency. In contrast, inhibition of Wnt signaling in cells undergoing reprogramming by small molecules, such as inhibitors of Wnt production (IWP2), significantly reduces the efficiency of somatic cell reprogramming [111,112]. In agreement, deletion or silencing of endogenous β-catenin diminishes the number of reprogrammed colonies [111,113]. Importantly, Ross et al. have shown that human fibroblasts from focal dermal hypoplasia (FDH) patients, carrying mutations in the *PORCN* gene and therefore defective in Wnt secretion, fail to reprogram to an induced pluripotent state. Defects in reprogramming of FDH fibroblasts can be rescued by ectopic activation of Wnt signaling or by overexpression of *PORCN* [114]. 

In order to investigate which specific Wnt ligand may be directly implicated during somatic reprogramming, Kimura et al. performed a population expression profile of cells enriched for undergoing reprogramming [115]. Their results proved that WNT2 is upregulated prominently during reprogramming simultaneously with increased nuclear β-catenin levels. Surprisingly, WNT2 is mainly expressed by cells that do not undergo reprogramming. This suggests that non-reprogrammed cells secrete positive signals, which support cells’ undergoing somatic reprogramming. 

Wnt pathway activation is one of the main factors implicated in maintaining mouse and human naive ESC pluripotency and therefore in the expression of the transcriptional pluripotent network. However, it has been shown that a reverse crosstalk from the pluripotency network towards the Wnt pathway exists, as well. NANOG, one of the main components of the ESC transcriptional core [116] and whose overexpression induces cell-fusion-mediated reprogramming [117], has a direct role in regulating the Wnt pathway [118]. NANOG binds to the *Dkk1* promoter and reduces its expression, which generates an increase in the levels of β-catenin and activation of its target genes. The crosstalk between NANOG and the Wnt pathway is essential for reprogramming since inhibition of the Wnt pathway in NANOG-overexpressing cells impairs the generation of reprogrammed cells [118].

In addition to Wnt proteins, timing and levels of Wnt activation during mouse somatic cell reprogramming influence the appropriate induction of the pluripotent state. Two independent reports have shown that, in order to achieve reprogramming, Wnt signaling must be repressed during the earliest stages of the process, while its activation is a requirement during the late steps of reprogramming [111,112]. Interestingly, sorting cells at early steps of the reprogramming process with no activation of the Wnt pathway significantly enriches the number of reprogrammed cells, underlining Wnt Off as an early reprogramming marker [111]. Furthermore, the threshold of β-catenin stabilization is very important for reprogramming efficiency. While complete absence of β-catenin stabilization inhibits somatic cell reprogramming, high or aberrant levels of β-catenin also have a negative effect on cell fusion-mediated reprogramming [103].

The central effect of β-catenin stabilization on rescuing the repressive influence of TCF3 on the self-renewal capacity of mouse ESC has stimulated the interest in studying the role of TCF/LEF factors in somatic reprogramming. Consequently, it has been demonstrated that in pluripotent stem cells, TCF1 and LEF1 act mainly as Wnt transcriptional activators, while TCF3 and TCF4 act as transcriptional repressors [23]. In accordance with the biphasic role of the Wnt pathway during reprogramming (Wnt Off at early events, Wnt-On during late events), silencing of TCF1 and LEF1 during the initial reprogramming steps and silencing of TCF3 or TCF4 in the later stages increases the efficiency of pluripotency induction [111,112]. In fact, absence of TCF3 expression, the main transcriptional repressor endogenously expressed in ESCs, has become a powerful tactic to increase cell fusion-mediated or factor-induced reprogramming even in the absence of overexpression of some Yamanaka factors as c-MYC and SOX2 [103,112].

However, the mechanism or the identity of the downstream reprogrammer(s) that is (are) activated by β-catenin/TCF signaling is still not entirely known. Increased levels of β-catenin by addition of CHIR99021 during induction of pluripotent stem cells or by overexpression of β-catenin in cell-fusion-induced reprogramming reduce cell proliferation, ruling out a pro-proliferative function of the Wnt pathway during somatic cell reprogramming [113,119]. Activation of the Wnt pathway increases the number of surviving cells undergoing cell fusion-induced reprogramming [119]. However, whether this is the main mechanism implicated in factor-induced reprogramming has not been yet investigated. A direct transcriptional target of the Wnt/β-catenin pathway, *c-Myc*, is one of the four Yamanaka factors capable of inducing reprogramming [120]. However, when activated in iPSCs or somatic cells undergoing reprogramming, the Wnt pathway either reduces or has no effect altogether on *c-Myc* expression [104,110,119], precluding a positive transcriptional effect on increased levels of *c-Myc* along reprogramming events.

TCF3 has been shown to repress the expression of several pluripotency-related genes. The absence of TCF3 opens the chromatin by increasing the levels of acetylated H3 and Histone 3 Lysine 4 trimethylation in both pluripotent cells and somatic cells undergoing reprogramming [121,122]. Furthermore, ablation of TCF3 increases the expression of pluripotency-related genes *Nanog*, *Esrrb*, *Dppa3* and *Zpf42*, among others [75,81,112]. Altogether, these results indicate that the positive role of the Wnt pathway and TCF transcription factors on somatic reprogramming is not based on regulating the expression of one or a few reprogrammers, but rather by establishing a chromatin state that allows the expression of several pluripotency genes needed to reach complete reprogramming.

In conclusion, several reports have already shown that even though Wnt/β-catenin pathway activation itself does not induce reprogramming to pluripotency; its endogenous autocrine–paracrine activity in somatic cells is necessary and enough to sustain cell fusion-dependent or direct reprogramming. External abolishment of somatic Wnt activity abrogates cellular reprogramming, and vice versa, activation of Wnt pathway increases reprogramming efficiency.

## 7. Conclusions and Future Directions

The canonical Wnt pathway plays major and critical roles during embryonic development of vertebrate organisms. An intricate control over its activity is required for the correct unfolding of the events that encompass the very beginning of life. One such role is governing the correct specification of the different cellular lineages, which will give rise to the different tissues and organs of the adult organism. Specifically, its activation drives differentiation and commitment to the meso-endodermal lineage during the post-implantation blastocyst stage. However, whether the Wnt signaling pathway also regulates the specification of extraembryonic tissues remains to be further studied. The importance of the Wnt pathway during the earliest stages of embryonic development remains unclear. Although the presence of β-catenin in the nucleus and expression of the Wnt target gene *Axin2* can be detected in the morula or pre-implantation blastocyst, transcriptional activity of the Wnt pathway seems to be dispensable for development until the blastocyst stage. This is demonstrated by the absence of developmental defects in β-catenin knockout (KO) pre-implantation blastocysts. To solve this paradox, further investigations should ensue, making use of embryonic stem cells.

Wnt signaling is essential for the maintenance of the homogeneous pluripotent naive state in ESCs. However, β-catenin KO ESCs maintain naive pluripotency in vitro as long as they are cultured in the presence of LIF. However, when deprived of this factor, β-catenin null cells rapidly exit naive pluripotency. On the other hand, inducing Wnt signaling in the absence of LIF supports the maintenance of the naive pluripotent state. As such, the Wnt and LIF pathways seem apparently redundant in the maintenance of naive pluripotency, and further studies should be performed in order to understand whether other pathways could be involved in the maintenance of in vitro naive pluripotency and early embryonic development in β-catenin KO mouse models.

Due to their ability to replicate indefinitely and their capacity to differentiate towards all embryonic lineages, iPSCs and ESCs hold a valuable potential for application in regenerative medicine. However, among other reasons, their use in regenerative therapies is not possible at the moment due to unamenable concerns regarding the tumor initiating capacity of pluripotent stem cells. The Wnt pathway has been recently shown to regulate both pluripotency and proliferation in mESCs. These cellular functions are seemingly regulated by different TCF/LEF Wnt-dependent transcription factors that govern discrete gene programs. This non-redundant nature of TCF/LEF transcription factors could potentially be exploited in order to achieve the modulation of particular beneficial Wnt-dependent cellular outcomes without concomitant modulation of undesired functions, i.e. maintaining iPSC/ESC self-renewal without oncogenic potential. As such, further investigation into the roles of Wnt signaling in the pluripotent cell cycle needs to be performed in the hopes of obtaining safer pluripotent stem cells, finally unlocking the grail of regenerative medicine.
